# Accelerating the Field of Epigenetic Histone Modification Through Mass Spectrometry–Based Approaches

**DOI:** 10.1074/mcp.R120.002257

**Published:** 2020-12-08

**Authors:** Congcong Lu, Mariel Coradin, Elizabeth G. Porter, Benjamin A. Garcia

**Affiliations:** 1 Department of Biochemistry and Biophysics, Perelman School of Medicine, University of Pennsylvania, Philadelphia, Pennsylvania, USA; 2 Epigenetics Institute, Perelman School of Medicine, University of Pennsylvania, Philadelphia, Pennsylvania, USA; 3 Biochemistry and Molecular Biophysics Graduate Group, Perelman School of Medicine, University of Pennsylvania, Philadelphia, Pennsylvania, USA

**Keywords:** histone post-translational modification, mass spectrometry, epigenetic regulation, middle-down proteomics, crosslinking MS, hydrogen–deuterium exchange MS, multi-omics, AKT, protein kinase B, AP, affinity purification, BIR, baculovirus IAP repeat domain, BS3, bissulfosuccinimidyl suberate, CBP, CREB-binding protein, ChIP, chromatin immunoprecipitation, CID, collision-induced dissociation, CL, crosslinking, CRISPR, clusters of regularly interspaced short palindromic repeats, DIPG, diffuse intrinsic pontine glioma, DSBU, disuccinimidyl dibutyric urea, DSS, disuccinimidyl suberate, DSSO, disuccinimidyl sulfoxide, EDC, 1-ethyl-3-(3-dimethylaminopropyl)carbodiimide hydrochloride, ERK, extracellular signal–regulated kinase, ETD, electron-transfer dissociation, EZH2, histone–lysine *N*-methyltransferase enzyme, FFPE, formalin-fixed paraffin embedded, HAT, histone acetyltransferase, hCYS, homocysteine, HDAC, histone deacetylase, HDX, hydrogen-deuterium exchange, HDMC, histone demethylases, HMT, histone methyltransferases, HSV-1, herpes simplex virus type 1, IP, immunoprecipitation, KDM5A, lysine-specific demethylase 5A, LC, liquid chromatography, MAPK, mitogen-activated protein kinase, MS, mass spectrometry, MSK, ribosomal protein S6 kinase, P300, histone acetyltransferase p300, PGC, porous graphitic carbon, PI3K, phosphatidylinositol 3-kinase, PTM, post-translational modification, PHD, plant homeodomain, RAF, RAF proto-oncogene serine/threonine-protein kinase, RAS, rat sarcoma, SAH, S-adenosylhomocysteine, SDA, succinimidyl 4,4'-azipentanoate, SILAC, stable isotope labeling by amino acids in cell culture, TCA, tricarboxylic acid, TGF-β, transforming growth factor beta, WCX-HILIC, weak cation exchange-hydrophilic interaction chromatography

## Abstract

Histone post-translational modifications (PTMs) are one of the main mechanisms of epigenetic regulation. Dysregulation of histone PTMs leads to many human diseases, such as cancer. Because of its high throughput, accuracy, and flexibility, mass spectrometry (MS) has emerged as a powerful tool in the epigenetic histone modification field, allowing the comprehensive and unbiased analysis of histone PTMs and chromatin-associated factors. Coupled with various techniques from molecular biology, biochemistry, chemical biology, and biophysics, MS has been used to characterize distinct aspects of histone PTMs in the epigenetic regulation of chromatin functions. In this review, we will describe advancements in the field of MS that have facilitated the analysis of histone PTMs and chromatin biology.

## Highlights

• Middle–down is the most suitable to study histone combinatorial post-translational modifications.
• Crosslinking MS has a variety of potential applications in exploring histone post-translational modifications.
• Hydrogen–deuterium exchange MS holds great promise to study the compaction of nucleosome.
• Multi-omics approaches are useful to study complex regulatory networks.

## Graphical Abstract

**Figure:**
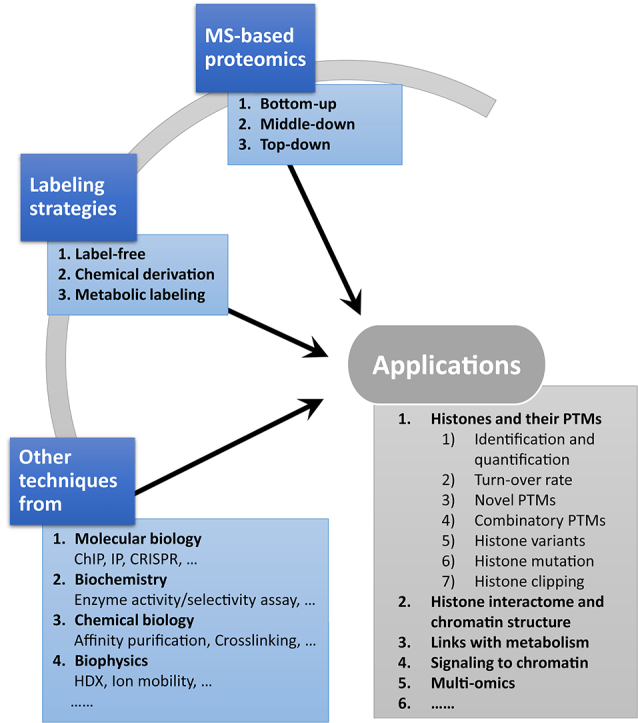

## In Brief

Histone post-translational modifications play essential roles in the epigenetic regulation of chromatin-related functions. Because of its high throughput, accuracy, and flexibility, mass spectrometry has emerged as a powerful tool in the epigenetic field. In this review, we describe the contributions of mass spectrometry–based proteomics in combination with distinct labeling strategies and various biological techniques to understand the roles of histone post-translational modifications and how they regulate chromatin function.

Chromatin acts as the instruction manual of eukaryotic cells. The nucleosome, the basic repeating unit of chromatin, is composed of ∼150 base pairs of DNA and two copies of the four core histones (H2A, H2B, H3, and H4) (1). Nucleosomes are then folded into more complex three-dimensional structures and dynamically remodeled by chromatin-binding proteins. The structure of the nucleosome has been well characterized by various techniques, providing insight into its roles in epigenetic regulation of chromatin function (2, 3). Epigenetics, the study of heritable gene regulation without alterations to the DNA sequence, can help determine gene expression, which influences pluripotency, cell-type differentiation, and responses to environmental changes. Well-known epigenetic regulation mechanisms include histone post-translation modifications (PTMs), DNA methylation, and noncoding RNAs (4, 5, 6).

**Fig. 1:**
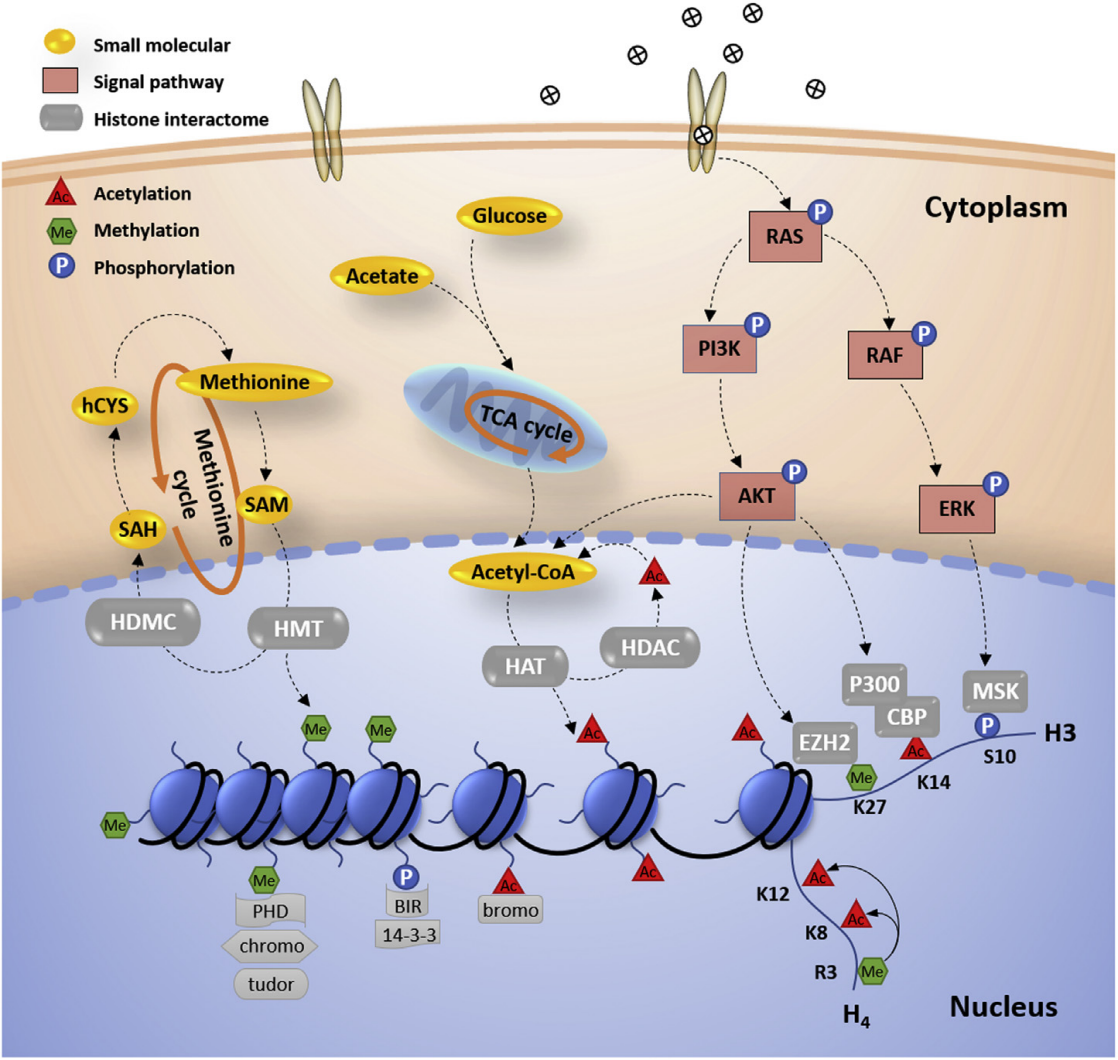
**Histone PTMs in epigenetic regulation.** (I) Two mechanisms by which histone PTMs regulate chromatin functions: (i) direct alternation of chromatin structure, *e.g.*, acetylation leads to an open chromatin; (ii) histone interactome, *e.g.*, plant homeodomain recognizes and binds to methylation and bromodomain to acetylation. (II) Three mechanisms influencing the expression of histone PTMs: (i) histone PTM crosstalk, *e.g.*, the presentence of histone H4R3me promotes the expression of H4K8ac and H4K12ac; (ii) metabolites as the precursors of modifications, *e.g.*, acetyl-CoA/acetylation, SAM/methylation; (iii) signaling pathways triggered by external stimuli, *e.g.*, PI3K/AKT and RAF/ERK modify histone H3 N-terminal tails. AKT, protein kinase B; BIR, baculovirus IAP repeat domain; CBP, CREB-binding protein; ERK, extracellular signal–regulated kinase; EZH2, histone–lysine *N*-methyltransferase enzyme; HAT, histone acetyltransferase; hCYS, homocysteine; HDAC, histone deacetylase; HDMC, histone demethylase; HMT, histone methyltransferase; MSK, ribosomal protein S6 kinase; PHD, plant homeodomain; RAF, RAF proto-oncogene serine/threonine-protein kinase; RAS, rat sarcoma; SAH, S-adenosylhomocysteine; TCA, tricarboxylic acid.

There are two main mechanisms by which histone PTMs exert epigenetic regulation (Fig. 1). The first involves modifications that directly influence the chromatin structure. For example, histone acetylation effectively reduces the positive charge of histones, weakening the interaction with negatively charged DNA, presumably leading to a less compact chromatin structure, thereby facilitating access to transcription factors and transcriptional machinery (7). The second one implicates PTMs in recruitment of chromatin-binding proteins. Numerous chromatin regulators recognize and bind specific histone modifications via distinct domains, such as plant homeodomain fingers, bromodomains, and chromodomains (8, 9, 10). There are at least three factors that affect levels of histone PTMs and further regulate chromatin functions (Fig. 1): (i) pre-existing histone PTMs that block or promote another modification via crosstalk (10), (ii) abundance of metabolite precursors of PTMs (11), and (iii) signaling cascade triggered by external stimuli (12).

**Fig. 2:**
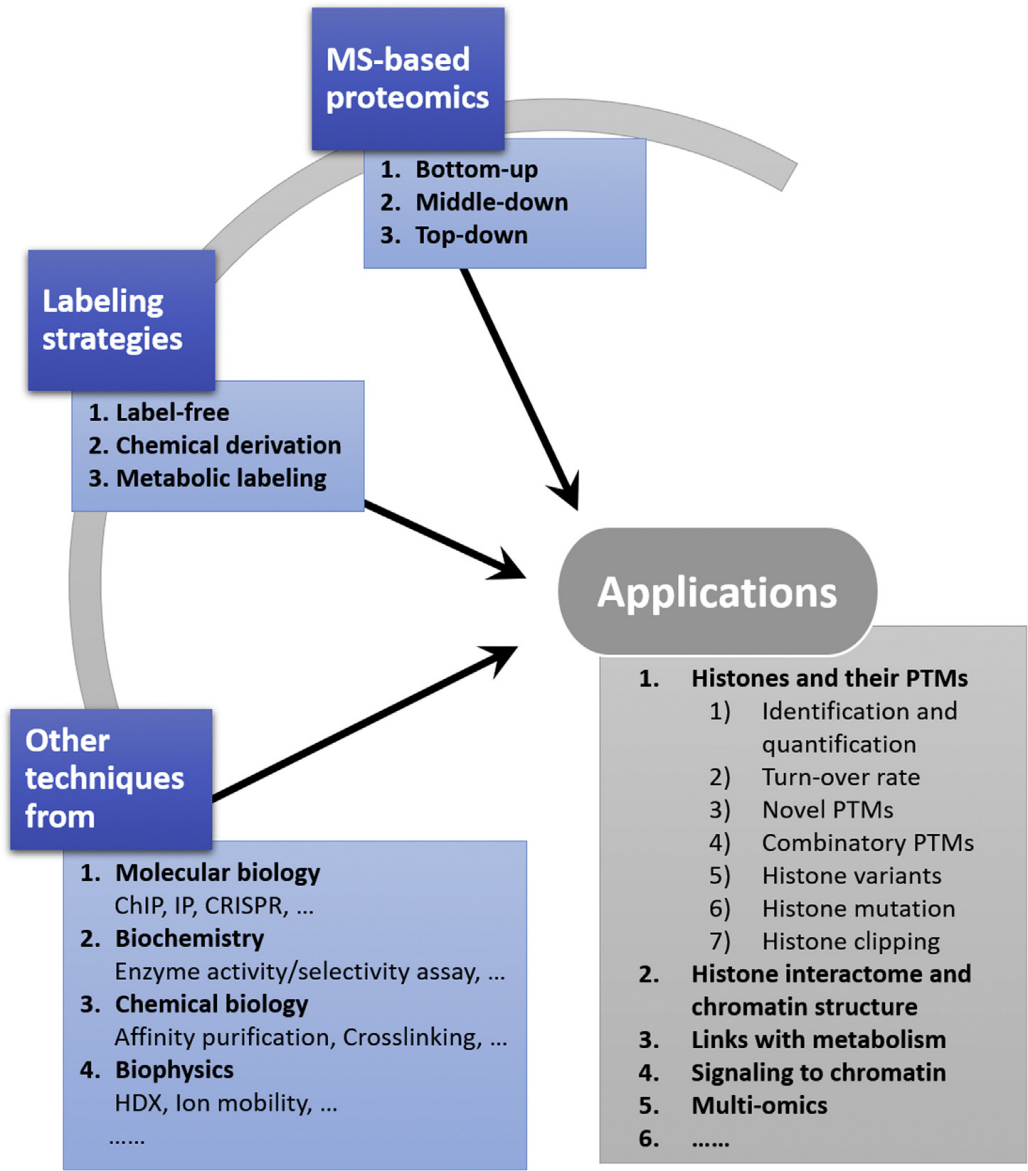
**MS-based proteomics approaches coupled with different labeling strategies and other techniques to understand mechanisms of histone regulation and chromatin functions.** ChIP, chromatin immunoprecipitation; CRISPR, clusters of regularly interspaced short palindromic repeats; HDX, hydrogen–deuterium exchange; IP, immunoprecipitation; MS, mass spectrometry; PTMs, post-translational modifications.

Increasing evidence shows that abnormal patterns of histone PTMs are associated with many human disorders, such as cancer, Alzheimer's disease, and autoimmune diseases (13, 14). Mass spectrometry (MS) has emerged as a powerful tool in epigenetic research field, allowing for the unbiased and comprehensive analysis of histone PTMs and the characterization of chromatin regulation on external stimuli. In this review, we will describe the contributions of MS-based proteomics in combination with distinct labeling strategies and various biological techniques to understand the roles of histone PTMs and how they regulate chromatin function (Fig. 2).

## Overview of MS-Based Proteomics Methods for the Analysis of Histone PTMs

**Fig. 3:**
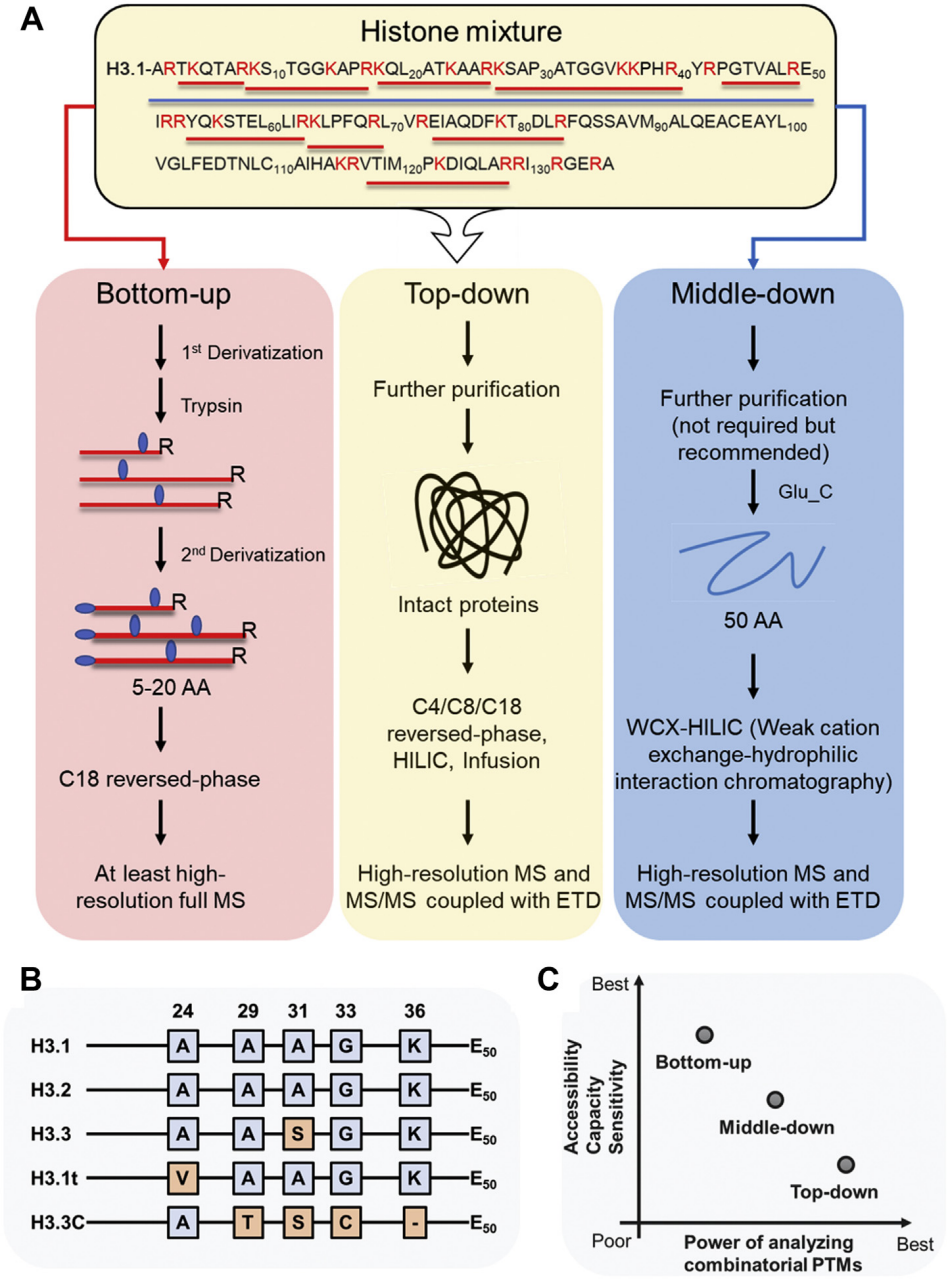
**MS-based proteomics analysis of histone PTMs.***A*, the most used workflow for proteomics analysis of histone PTMs (use H3 as example, lysine and arginine residues are labeled in *red*. Peptides with *red* underline are sent for bottom–up and *blue* for middle–down analysis). *B*, N-terminal tail sequence differences (1–50 AA) between H3 variant representatives. *C*, comparison of three proteomics approaches in analyzing histone PTMs. ETD, electron-transfer dissociation; MS, mass spectrometry; WCX-HILIC, weak cation exchange-hydrophilic interaction chromatography.

There are three MS-based proteomics methods that can be used for the analysis of histone PTMs: bottom–up, middle–down, and top–down approaches (Fig. 3) (15, 16, 17). Sample preparation is identical up to the point where histones are extracted. Briefly, cell pellets or frozen tissues are homogenized into a hypotonic lysis buffer, removing the cytoplasm while the nuclei remain intact. Histones are then purified using acid extraction followed by precipitation with trichloroacetic acid (18, 19).

### Bottom–up Proteomics

The core histone proteins (H2A, H2B, H3, and H4) are known to be relatively small, ranging from 10 to 15 kDa. Unstructured tails of the core histones project outward from the nucleosome and are heavily post-translationally modified, controlling the epigenetic regulation of the genome. Histones are also very basic proteins, containing an overwhelming number of trypsin cleavage sites (lysine and arginine residues, Fig. 3*A*). In a typical bottom–up experiment, proteins are digested with trypsin. However, use of trypsin on histones results in peptides that are too short to retain on reversed-phase chromatography columns and a lack of charge density, making them not amenable for MS. Thus, derivatization of free amine groups on the N termini and lysines before a trypsin cleavage is frequently applied to mimic an Arg-C digestion while using the robust enzyme trypsin.

Different derivatization methods have been optimized in several laboratories, from the use of formaldehyde (20) to maleic anhydride (21) to propionic anhydride (22). Sidoli *et al*. (23) tested 12 commercially available anhydrides for histone derivatization and concluded propionic anhydride derivatization as the best to achieve high MS sensitivity and ionization efficiency for histone analysis. In addition to preventing cleavage at lysine residues, the propionic anhydride derivatization neutralizes the charge, making the resulting peptides less hydrophilic. The peptides can now be easily resolved by standard reversed-phase chromatography. Stable isotope-labeled anhydrides can be introduced in the second derivatization step after trypsin digestion to label newly generated peptide N termini, allowing accurate quantification and comparison of histone PTMs from two separate conditions in one MS run (20, 22).

The protocol for bottom–up analysis has been optimized for histone extraction (18), derivatization (24, 25), MS acquisition method, and data analysis to provide the most robust and accurate characterization of histone PTMs (26, 27). Data-dependent acquisition remains the most common MS data acquisition method in proteomics, whereas data-independent acquisition is now widely used in histone bottom–up proteomics to discriminate and quantify relative abundance of coeluting isobaric peptides like H3K18ac and H3K23ac based on the profile of the fragment ions (28, 29, 30).

### Top–Down Proteomics

Contributing to the complexity of chromatin regulation, histone PTMs rarely function independently and are often found in combination. Pre-existed histone PTMs can influence whether another modification will be deposited, which is known as crosstalk (Fig. 1). Distinct patterns of histone PTMs distinguish chromatin elements and recruit the proper regulatory proteins to those regions (31). Together, these constitute the ‘histone code’ regulation mechanisms (8, 10). The traditional bottom–up method with chemical derivatization generates short tryptic peptides (5–20 AA), which are not suitable for the study of combinatorial PTMs, especially for crosstalk between long-distance modified sites such as H3K4 to H3K27. Other workflows, namely top down and middle down, are used to quantify the combinatorial histone PTMs (32, 33).

Top–down approach analyzes intact histone proteins, and no proteolytic digestion is performed prior to MS analysis (Fig. 3). This method requires high-resolution MS and MS/MS because of the high charge state of intact histones (ranging from 13+ to 25+ for H3) (32). The main challenge is the large number of potential proteoforms that cannot be resolved by HPLC–MS/MS. For instance, 31 modifiable sites have trillions (10E12) of theoretical proteoforms (34). Multiple isoforms can coelute and cofragment, decreasing detection sensitivity and making identification of MS2 highly challenging. Top–down approach is also computationally challenging (16). Only few computational programs have been adapted to analyze heavily modified histone proteins, and even fewer tools exist to go from identification to quantification. Because of this, histone analysis via top down remains sparsely used for large-scale analyses, but some improvements have increased the sensitivity and capabilities and are encouraging for the future of top–down strategy (28). Two comprehensive reviews by Neil Kelleher's laboratory, one of the leading research groups in the top–down field, have described the history and fundamental concepts of top–down proteomics and discuss recent technical and conceptual advances (35, 36). We strongly suggest readers intending to learn more about this methodology to use these two reviews as a resource.

### Middle–Down Proteomics

As a compromise between bottom–up and top–down proteomics, middle–down proteomics is used to analyze long polypeptides that retain more information about coexisting PTMs than bottom–up proteomics and is far less technically challenging than top–down proteomics. This approach has proved to be of high throughput and feasible, reaching the similar level of reliability as bottom up (37). A detailed discussion of the basic principles of middle–down analysis of histone PTMs is not included here, as much, we refer the interested readers to specialized reviews (15, 38).

The most commonly used proteases in histone middle–down analysis are Glu-C for histone H3 (generating 1–50 AA) and Asp-N for H4 (generating 1–23 AA), as the resulting polypeptides carry the most prevalently studied modification sites. Histone N-terminal tails are separated through a hybrid weak cation exchange-hydrophilic interaction chromatography and analyzed by high-resolution MS and MS/MS with electron-transfer dissociation fragmentation to reach the maximum detection sensitivity. However, because of the high complexity of this method, *e.g.*, special chromatography with a different buffer system compared with reversed-phase liquid chromatography (LC) and relative complex data processing steps, there are very few chromatin biology studies using middle–down approach (38). Coradin *et al*. (39) recently described critical aspects of middle–down proteomics and provided a streamlined way to evaluate its performance in identification and quantification of histone combinatorial PTMs, which is a useful resource for beginners.

Reversed-phase chromatography is a more robust type of chromatography than weak cation exchange-hydrophilic interaction chromatography, and it is convenient for proteomics laboratories that have C18 columns coupled with MS. However, it has far less efficient separation and thus leads to less-sensitive results. Several attempts have been made to make reversed-phase chromatography more suitable for middle–down analysis. Liao *et al*. (40) described a chemical derivatization strategy that improves sensitivity for profiling combinatorial histone PTMs using reversed-phased C18 column. Schräder *et al*. (33) used an alternative protease, neprosin, to generate H3 peptide 1 to 38 AA as the most abundant H3 digestion product, which allows better retention and dispersion than H3 N-terminal tail 1 to 50 AA on reversed-phase chromatography. Janssen *et al*. (41) used porous graphitic carbon as a novel stationary phase and achieved simultaneous bottom–up and middle–down histone analysis using the same reversed-phase buffer setup. More recently, ion mobility has been applied to separate isobaric forms that cannot be resolved by LC (42, 43, 44), paving new ways using orthogonal methods to improve middle–down sensitivity.

## Analysis of Histones and Their PTMs

The high throughput, accuracy, and flexibility of MS make it the most suitable strategy for the comprehensive analysis of histone PTMs. Although characterization of novel histone PTMs is most frequently performed by MS, quantification or validation is still performed by either or both antibody-based and MS-based techniques. Both have advantages and certain limitations (34). They work independently or in combination, providing critical roles in analyzing histone PTMs.

### Antibody-Based Techniques

Analysis of histone PTMs and their genomic locations have heavily relied on antibodies. Investigation by methods such an immunoblotting and immunofluorescence is common in most biological laboratories and do not require expensive instrumentation such as nanoLC–MS/MS or complex workflows. There is a plethora of commercially available antibodies that recognize the different histones and their variants as well as residue-specific modifications, even disgusting between methylation states.

Histone antibodies are often used for chromatin immunoprecipitation (ChIP), a method that isolates a protein along with the chromatin it associates. This can be further coupled with next-generation sequencing (ChIP-Seq) to map genomic regions where individual histone modifications are localized (45, 46). These experiments have greatly advanced our understanding of the link between histone PTMs and *cis* regulatory elements, providing information that is not available by MS-based methods. Efforts such as the Encyclopedia of DNA Elements project have worked to create a database of the localization of many histone modifications in commonly used cell lines (47). The basic principle of ChIP has also been coupled with other techniques such as DNA barcoding and MS. Weiner *et al*. (48) developed combinatorial indexed ChIP for analyzing combinatorial marks. In this method, nucleosomes are isolated with a particular antibody and labeled with a DNA barcode followed by disassociation from the antibody. The barcoded histones are then pooled and incubated with a different antibody, allowing for the identification of coexisting PTMs. ChIP can also be adapted to prepare samples for MS analysis (ChIP-MS), allowing for a broader interrogation of the state of chromatin (49). After immunoprecipitation, the proteins rather than the DNA can be isolated and characterized by MS.

A major limitation to antibody-based techniques is the efficiency and specificity of the antibody. Antibody crossreactivity is commonly observed with different modification states on the same residue (*e.g.*, me1, me2, or me3) or the same modification at different residues with similar sequence motif (*e.g.*, Ala-Arg-Lys-Ser sequence at H3K9 and H3K27). Egelhofer *et al*. (50) tested the specificity of more than 200 antibodies against 57 histone modifications and observed that more than 25% of commercial antibodies fail specificity tests by dot blot or Western blot, and among specific antibodies, more than 20% fail in ChIP experiments. To complement the Antibody Validation Database built by Egelhofer *et al*., which offered the demonstration of an antibody's ability to work or not in Western blot or ChIP, Histone Antibody Specificity Database was created to demonstrate the actual specificity and crossreactivity of more than 100 commercial PTM-specific antibodies (51). Both databases facilitate important antibody information for epigenetic applications and greatly aid researchers in making more informed histone antibody choices.

### MS-Based Techniques

MS-based method provides the most unbiased quantification and highest throughput in a single analysis. Recently, Sidoli *et al*. (52) achieved 1-min MS quantitative data on ∼200 histone PTMs via direct injection using bottom–up MS. This new protocol takes 7 h from start (cell pellets or tissue) to finish (data analysis), providing the possibility of super high-throughput screening of >1000 samples per day. Furthermore, histone PTM dynamics, turnover, and/or stoichiometry in biological systems can be investigated using metabolic labeling and analyzed by middle–down or bottom–up MS (53, 54, 55, 56).

In addition to analysis of previously characterized PTMs, MS has also emerged as a fundamental tool in categorizing novel modifications. For example, Yingming Zhao's group identified nine distinct types of short-chain lysine acylations (butyrylation, crotonylation, glutarylation, lactylation, malonylation, propionylation, succinylation, β-hydroxybutyrylation [also called 3-hudroxybutyrylation], and 2-hydroxyisobutyrylation) (57, 58, 59, 60, 61, 62, 63, 64), vastly expanding the list of known modifications found on histones. Because of the low abundance of these PTMs, acylations are often immune-affinity enriched using pan- or site-specific antibodies before MS or genomic analyses.

In addition to antibody-based enrichment methods, chemical probe–based strategies are also used for histone modification enrichment and/or detection. For example, Li's laboratory designed chemical reporters to specific enrich malonylation or glutarylation (65, 66). The Muir laboratory developed chemical probes for crotonylation or serotonylation (67, 68). Compared with immunoblotting methods, chemical probes are more ideal for monitoring the modification dynamics (65). These probes are less likely to have off-target interactions, preventing nonspecific interactions common in immunoblotting methods. They can also be linked to biotin, allowing for efficient and clean isolation of a modification from whole cell lysate using streptavidin resin (67). However, the probes do not provide residue-specific information like antibody methods and usually require a complex enrichment workflow.

Characterizing and validation of histone PTMs as well as quantification using MS are accurately explained by Huang *et al*. (69). Altogether, more than 500 modifications have been characterized on histones. For a comprehensive catalog of histone PTMs and their corresponding functions, we refer readers to the reviews listed (70, 71).

### Middle–Down Proteomics Applications

Research of histone PTMs was propelled by the proposal of the histone code hypothesis in 2000 (8). The histone code hypothesizes that DNA transcription is regulated sequentially or in combination by distinct histone PTMs. Beyond PTMs, histone variants can also alter gene expression patterns. Histone variants have similarities from their canonical counterparts. The differences between variants and canonical histones can be changes of a couple amino acid (Fig. 3*B*) (72) to the addition of a 30 kDa domain (73). There are multiple variants for all the canonical variants besides histone H4. Histone variants have distinct expression patterns, and region-specific distribution, providing alternative mechanisms in maintaining transcriptional regulation, DNA repair, and other cellular processes (74, 75). Adding to their general role in transcription, mutations in the genes encoding histones have been discovered and linked to cancer. These mutant histones have been termed oncohistones because their expression shapes an oncogenic transcriptome and have recently been linked to cancers (14, 76). The mostly well-characterized mutation that MS revealed is H3K27M. The mutation represents 3 to 17% of total H3 in diffuse intrinsic pontine glioma patient cells and has been seen to disrupt polycomb repressive complex 2 function (77). New layers of regulation have also been discovered such as proteolysis or clipping of histone tails, where the N-terminal tail is irreversible cleaved by intracellular proteases. This phenomenon has been observed in multiple organisms and regulates many biological functions (78, 79, 80) such as regulation of differentiation of human stem cells (81) and has been observed in human cancer (82).

Although great progress has been made, many questions remain unanswered, especially the characterization and functional analysis of combinatorial histone PTMs, histone variants, histone mutation, and histone clipping. These questions can hardly be investigated with bottom–up proteomics, and other techniques have not been optimized to answer these questions. Middle–down and top–down techniques remain scarcely exploited in chromatin biology studies because of the high complexity of the method. As described in the previous part, middle–down technique is a suitable compromise between bottom–up and top–down proteomics, allowing for the analysis of histone N-terminal tails where most PTMs reside. Increasing number of studies has successfully proved efficacy of middle–down technique in unraveling crosstalk between histone PTMs (56, 83, 84). Middle–down technique also allows for discrimination of highly similar histone variants as the sequence variations are usually present in the N-terminal tails. In addition, it can also be used to detect histone clipping because of the protease-specific cleavage of histone tails, indicating all other fragments would be the result of clipping. Although it has certain limitations, middle–down technique has been conducted on histone PTMs, elucidating unexplored biological aspects that are challenging to illuminate by bottom–up proteomics (56, 84).

## Histone Interactome and Chromatin Structure

The addition of small chemical moieties on histone proteins, *e.g.*, acetylation and phosphorylation, effectively reduce the positive charge of histones, influencing the overall structure of chromatin, facilitating or impairing DNA access (7, 85). This is one of the two main mechanisms by which histone PTMs regulate chromatin functions. The other one involves numerous chromatin-associated factors that specifically interact with modified histones. The histone interactome is popularly categorized into writers, readers, and erasers, where writers and erasers modify histones by catalyzing the addition and removal of PTMs, whereas readers recognize and translate these PTMs (Fig. 1). So far, there are more than 50 different histone writers and erasers identified, including kinases, histone acetyltransferases, histone deacetylases, histone methyltransferases, and histone demethylases. Similarly, the number of known histone readers is large. Their classification and functions are comprehensively summarized in many reviews (9, 85, 86).

### Affinity-Purification MS

Unraveling the histone interactome is key to not only reveal how one PTM affects the levels of another one but also disentangle how histone PTMs translate into a physiological response. Different enrichment protocols have been proposed in the last decades, but a gold standard method does not exist. The most widely used method is the one that uses tagged peptides containing the PTMs of interest to isolate and detect interacting proteins by affinity-purification MS (AP-MS) (87). Similar approaches using small molecules, DNA, and nucleosomes to affinity purify target proteins for MS analysis has been applied to extend the histone interactome (49, 88, 89, 90, 91). Noberini *et al*. (92) recently reviewed 17 AP-MS–based strategies to dissect chromatin-associated proteins.

Many histone-binding factors are transiently associated with chromatin or present at low abundance, making it difficult to discover these interactions. Few approaches are used for accurate and sensitive detection of histone-binding factors. One strategy combines quantitative proteomics with AP-MS. In conjunction with stable isotope labeling by amino acids in cell culture, Vermeulen *et al*. (93, 94) identified readers of the activating marks H3K4me3 and H3K36me3 and silencing marks H3K9me3, H3K27me3, and H4K20me3 using a peptide pull-down approach. Another method takes advantages of crosslinking (95). Crosslinkers are introduced to convert dynamic and transient binding to irreversible covalent linkages, minimizing the proteins loss and allowing for more stringent washes to reduce nonspecific bindings. For example, Li *et al*. (91, 96) designed a probe with histone H3K4me3 for recognizing modification-binding proteins, a photocrosslinker group for capture the PTM binders, and an alkyne group for rapid detection and affinity enrichment through click chemistry. The aforementioned two methods can be used together. For instance, Kleiner *et al*. (97) used photocrosslinking and stable isotope labeling by amino acids in cell culture–based proteomics for profiling histone H3 and H4 interactome.

### Crosslinking MS

**Fig. 4:**
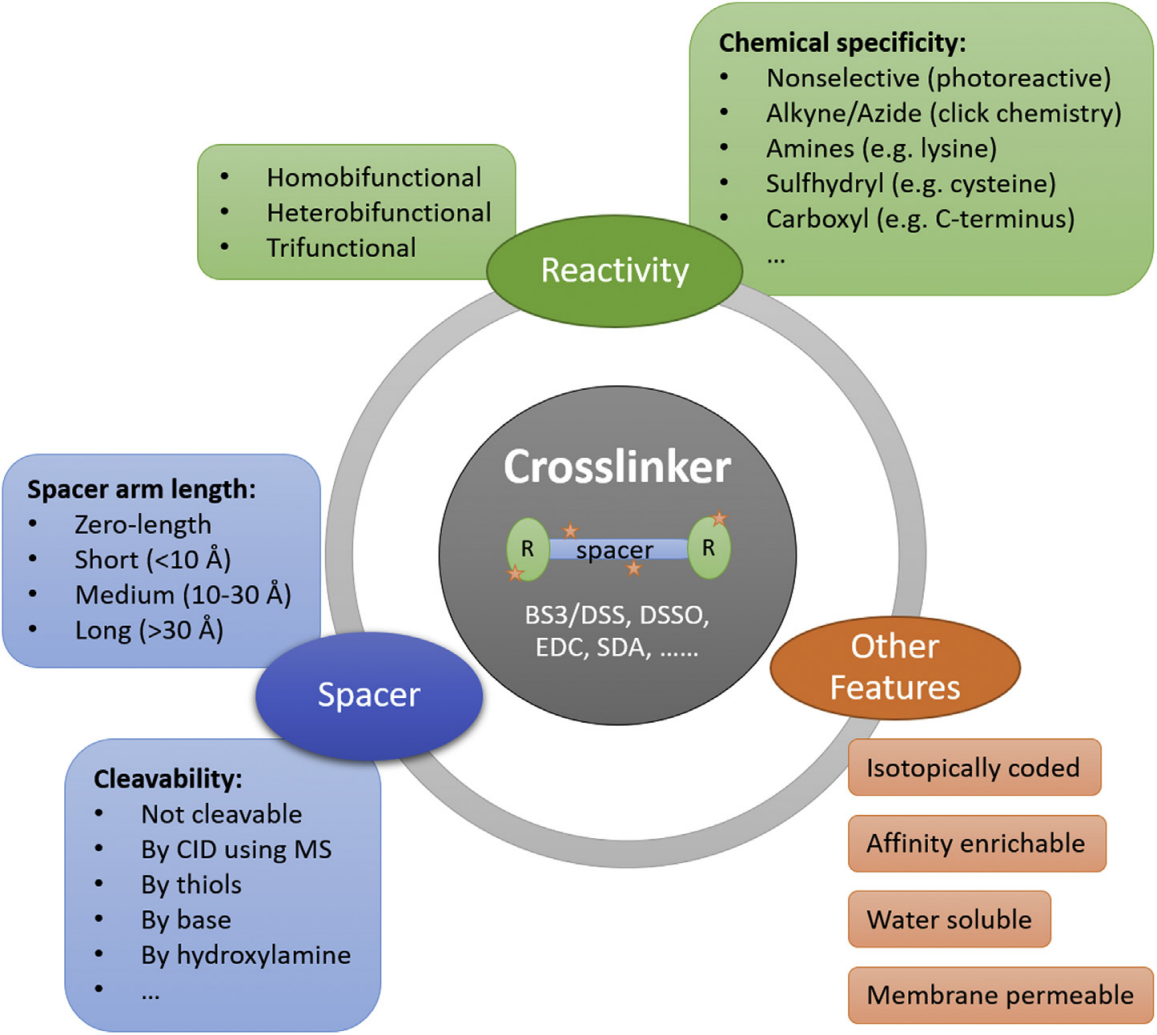
**Crosslinker features to consider for certain MS-based experiments.** BS3, bissulfosuccinimidyl suberate; CID, collision-induced dissociation; DSS, disuccinimidyl suberate; DSSO, disuccinimidyl sulfoxide; EDC, 1-ethyl-3-(3-dimethylaminopropyl)carbodiimide hydrochloride; MS, mass spectrometry; SDA, succinimidyl 4,4'-azipentanoate.

Crosslinking MS (CL-MS) can not only derive protein relationships for biological networks but also shed light on medium-resolution structure analysis (98, 99). Inefficient detection of crosslinked peptides within the complex tryptic mixtures has been a long-standing bottleneck in CL-MS. Recent developments of novel crosslinkers with multiple features (Fig. 4), enrichment of crosslinked peptides using strong cation exchange, and database search algorithms (*e.g.*, XlinkX, MeroX, pLink, MetaMorpheus) vastly improved the sensitivity and complexity of CL-MS (100, 101, 102). False discovery of cross-linked peptides remains a challenge in CL-MS although a lot of progress has been made in the algorithms (103). Beveridge *et al*. (104) measured the actual false crosslink identification rates of the most frequently used search engines using a synthetic peptide library, and they found that the rates range from 2.4% to 32% depending on the analysis strategy used.

Although CL-MS now can be achieved at proteome-wide providing interactions that cannot be detected by AP-MS (105, 106), there are only limited studies using CL-MS to analyze nucleosome-interacting proteins and barely discussed histone PTM interactome (107, 108). One of the central obstacles is that histones are rich in lysine residues, which react with amino-specific crosslinkers and can be cleaved by trypsin. Furthermore, histones are heavily modified, which brings more challenges to the computational analysis. Improvements would be necessary for novel crosslinkers, alternative proteases, and software to better apply CL-MS in histone-related studies.

### Hydrogen–Deuterium Exchange MS

Although the structure of the nucleosome core has been crystallized and solved, many questions remain about the N-terminal tails. Because of the dynamic and flexible nature of the tails, they are challenging to crystallize. Hydrogen–deuterium exchange MS (HDX-MS) is an emerging technique that will be able to elucidate these and similar questions in the structural biology field. It has been used to locate protein sites that are directly or indirectly involved in binding, monitor the folding dynamics of protein domains, and characterize nuclear receptor complex ligand regulation (109). Compared with classical techniques for structural analysis like X-ray crystallography, HDX-MS does not require crystallization or high-purified samples and can be used to investigate structural dynamics under native solution-phase conditions with small amount of materials (110). In 2013, D'Arcy *et al*. (111) used HDX-MS to map the binding interface between H2A/H2B dimer and its chaperone nucleosome assembly protein 1. More recently, Karch *et al*. (112) combined HDX with top–down and middle–down proteomics to study histone dynamics before and after nucleosome assembly. They found that the H4 tail is significantly protected in the nucleosome compared with the (H3/H4)_2_ tetramer.

Although incredible progress has been made in chromatin structure and tremendous numbers of histone-binding proteins have been detected, we are far from fully understanding the regulation mechanisms of histone PTMs in chromatin functions. The flexibility of MS makes it feasible to couple with various techniques from molecular biology, biochemistry, chemical biology, and biophysics, facilitating the studies of how histone PTMs regulate gene expression and DNA-related functions.

## Impact of Metabolism on Histone PTMs

So far, we have discussed the applications of MS in characterizing histones and their PTMs from distinct aspects, including PTM crosstalk, binding partners, and structural analysis. In this section, we will discuss how the abundance of metabolites that are precursors influence histone PTM levels (Fig. 1, *e.g.*, acetyl-CoA/acetylation and SAM/methylation) and how this is monitored by MS.

Increasing evidence proves that the addition of histone marks, in particular histone acylation (11), can be both enzymatic and nonenzymatic in nature. Using quantitative MS, Simithy *et al*. (113) observed a dose-dependent increase of histone acyl-PTMs (acetyl, propionyl, butyryl, crotonyl, malonyl, succinyl, β-hydroxybutyryl, and glutaryl) abundance in response to the concentration of acyl-CoA without enzyme catalyzing *in vitro* and *in vivo*, indicating that the level changes of precursor metabolites directly alter the levels of histone PTMs.

As mentioned before, growing cell cultures in the presence of stable isotope–labeled metabolites enables metabolic tracing and turnover rate of histone PTMs. Zhang *et al*. (64) used [13C_3_]lactate to demonstrate that a newly identified histone lactylation is derived from lactate. Kori *et al*. (114) observed preferential utilization of glucose for acetylation over acetate by heavy isotope labeling of either glucose or acetate. Mentch *et al*. (54) performed cell culture experiments in conditional media with or without methionine, the precursor of the methyl donor SAM, and detected rapid changes in H3 methylation. Sidoli *et al*. (56) used the heavy [13CD_3_]methionine and analyzed histone combinatorial PTM dynamics during epithelial to mesenchymal transition via middle–down MS.

Increasing sensitivity and specificity of MS-based methods has resulted in the identification of multiple novel histone modifications, particularly histone acylations (71). Acyl-CoAs are the precursors of histone acylations. They are generated in diverse metabolic processes, including amino acid catabolism, lipid metabolism, and ketone body metabolism. Unlike acetylation, where its transport out of mitochondria is well established, most production pathways of acyl-CoA in the nucleus are poorly understood (115). Data so far suggest that each acyl-CoA species have distinct roles in metabolism, and the corresponding histone acylations have been implicated in cell differentiation as well as other important biological processes. For example, histone lactylation increases under tumor-associated M1 macrophage polarization (64). Recent evidence supports that epigenetic changes in cancer could be driven by altered cellular metabolism (116), and the altered epigenetic landscape can affect the expression of genes involved in cell metabolism, presenting a complex regulatory network (12, 117). However, many unanswered questions remain regarding the carbon sources that contribute to histone lysine acylations and the mechanisms of how metabolites serve as signals linking metabolism to transcriptional responses.

## Signaling to Chromatin

Epigenetic responses to environmental changes lead to alterations in cell phenotype and cell fate decisions. External messengers activate cell receptors, subsequently triggering a series of downstream signalings to the nucleus that regulate gene expression patterns. In this section, we discuss the third factor that influences expression of histone PTMs, signaling cascade triggered by external stimuli (Fig. 1).

Most signaling cascades use phosphorylation as messengers. There are more than 200,000 known human phosphosites listed in PhosphoSitePlus and PhosphoNET, which modify more than two-thirds of proteins in the human proteome (118). High-throughput MS-based proteomics is ideally suited for such large-scale studies. Olsen *et al*. (119) achieved the first global *in vivo* and site-specific phosphorylation dynamics in signaling using MS-based quantitative proteomics in 2006. More progress has been made since then, from phosphopeptides enrichment to ion fragmentation and computational tools (120), providing deep insight into the molecular signaling and regulatory networks. More detailed analyses of the molecular architecture of signaling in different biological systems are provided elsewhere (12, 121).

Although the signaling mechanisms have been well studied, the interconnection between signaling cascades and chromatin to regulate epigenetic features is not fully understood. Single-dimensional phosphoproteomics analysis could not answer this question. Kulej *et al*. (122) performed a multidimensional analysis during herpes simplex virus type 1 infection, which includes proteome, phosphoproteome, chromatin-enriched proteome, and comprehensive analysis of histone PTM dynamics, providing unprecedented resolution and comprehensive proteomic data sets to guide future studies. Lu *et al*. (123) used a time-resolved epiproteomic approach to study epithelial to mesenchymal transition process, which featured the identification of the correlation between protein changes (proteome), transforming growth factor beta signaling pathways (phosphoproteome), and chromatin modulation (histone modifications). Taken together, these studies highlight the importance of MS-based approaches to understand biological processes. Moreover, the integration of proteomics, phosphoproteomics, and epigenomics can elucidate complex signaling mechanism. In the next section, we will discuss how multiomics approaches provide a holistic understanding of epigenetic regulation.

## Multiomics Approach to Study Chromatin Biology

Numerous signaling pathways have been characterized in different biological processes. Other than one-dimensional cascades, how cells response to external stimuli is controlled by a complex and intertwined regulatory network on multiple levels (12). For instance, phosphatidylinositol 3-kinase–AKT (protein kinase B), one of the core oncogenic signaling pathways, may directly phosphorylate histone modifiers (*e.g.*, histone acetyltransferase p300, histone methyltransferase histone–lysine N-methyltransferase enzyme [EZH2], and histone demethylase KDM5A [lysine-specific demethylase 5A]) to promote transcriptional regulation (121). On the other hand, AKT downstream signaling facilitates the metabolic production of acetyl-CoA (124), which is the precursor of acetylation. Dysregulation of this pathway could lead to abnormal allocation of acetyl-CoA, directly influencing histone acetylation levels (11). Moreover, the epigenetic regulation by histone PTMs can affect the expression of genes in other pathways and cell metabolism.

**Fig. 5:**
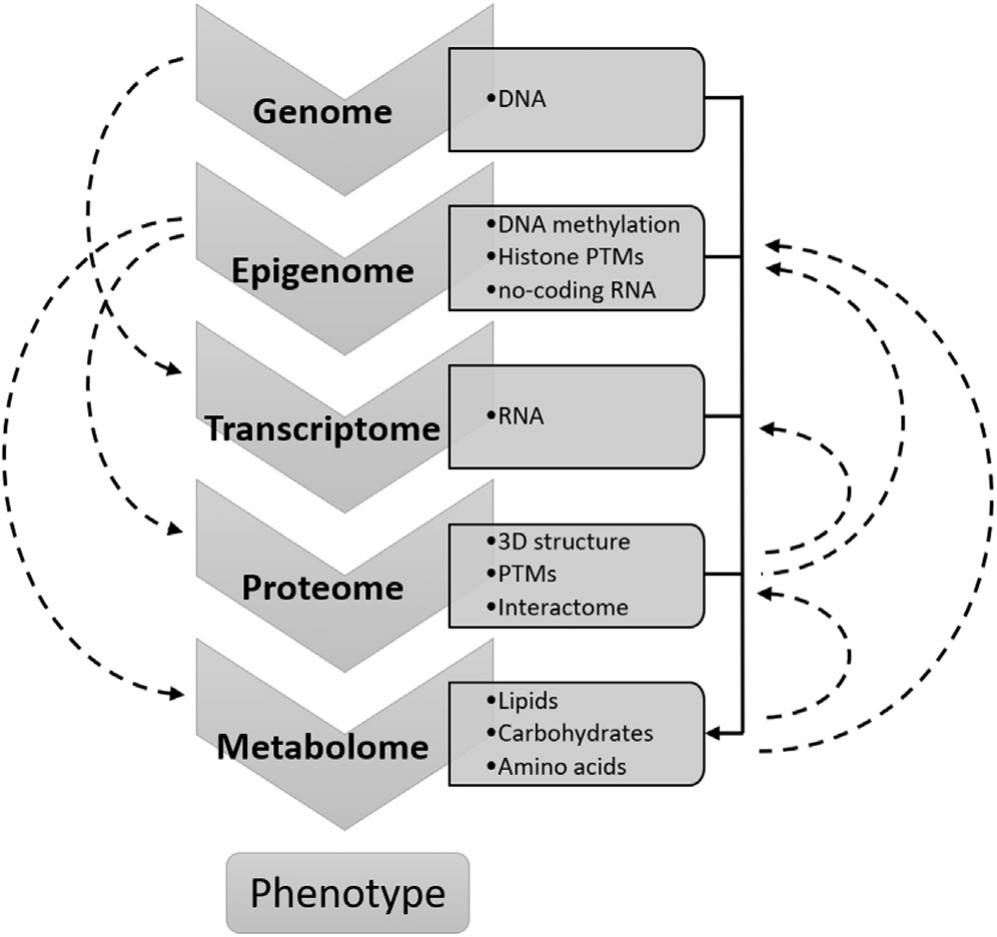
**Multiomics data types and their connectivity.** For instance, epigenome can change the expression of transcriptome, proteome, and metabolome. On the other hand, epigenome can be modified by proteome and metabolome. PTMs, post-translational modifications.

In the past decade, high-throughput omics technologies have revolutionized chromatin biology studies, especially in medical and clinical fields. Compared with studies of a single omics type, multiomics offers the integration of different data types, including genome, epigenome, transcriptome, proteome, metabolome, and sometime microbiome (Fig. 5). Together, these data are used to understand the molecular patterns associated with disease development such as cancer (125). For example, Backman *et al*. (126) combined transcriptome, proteome, and metabolome to study the key drivers of functional alterations of liver in insulin-deficient diabetes mellitus. Huang *et al*. (127) profiled global histone PTMs across 83 cancer cell lines with various sensitivities to EZH2 inhibitor. EZH2 is the writer of the epigenetic mark H3K27me3, which is associated with transcription repression. In this study, they detected different expression levels of MLL1 (histone-lysine N-methyltransferase 2A) between sensitive and insensitive cells using quantitative proteomics analysis. MLL1 is known to be the writer of H3K4me3. This mark is usually found in active genes. Transcriptomics, proteomics, and phosphoproteomics analyses demonstrated that H3K27ac upregulation (facilitated by MLL1 on EZH2 inhibitor treatment) lead to the activation of oncogenic signaling pathways like mitogen-activated protein kinase (also known as extracellular signal–regulated kinase) (127). These studies demonstrated that the integration of multiomics approaches can reveal mechanistic processes regulated by chromatin networks in diseases.

## Perspectives

Histones PTMs, together with DNA methylation and noncoding RNA, play essential roles in the epigenetic regulation of chromatin-related functions, ultimately defining the cell fate and transcriptional outputs of differentiated cells. Histone PTMs occur not only in the N-terminal tails but also on the lateral surface of the nucleosome (128, 129). Different from the N-terminal tail domains, which project out of the nucleosome, the lateral surface is directly in contact with DNA and formed by the histone core domains. The N-terminal tail PTMs have been well studied during the past decades; however, less is known about the core domain, requiring future studies on lateral modifications. Meanwhile, considering the diversity of potential acyl groups in the acyl-CoA-ome, there are almost certainly more acylation marks derived from metabolism, which could modify histones and regulate gene expression. Regarding the newly identified short-chain lysine acylations, a deeper investigation of carbon sources and key enzymes will help to illuminate the metabolic pathways that connect histone modification to metabolism as well as understand the functions of histone PTMs in chromatin biology.

MS-based proteomics has a variety of potential applications that could unravel unexplored histone PTM functions in chromatin regulation from different contexts. The possibilities of middle–down proteomics are yet to be fully exploited in chromatin regulation studies (38). Although it has certain limitations, middle–down technique is the most suitable one to study histone combinatorial PTMs, histone variants, mutations, and clipping. We speculate that middle–down MS will be popular and potentially replace the bottom–up MS in histone analysis in the near future as it can provide more layers of information. CL-MS is an emerging tool to aid in interactome research and structural biology and now is a robust and flexible technique with optimized and feasible workflow (97, 99). Although currently not optimal with histone-related studies, as work continues, CL-MS has a variety of potential applications that could unravel unexplored histone PTM aspects. Modified nucleosome now can be generated through multiple strategies (130), *e.g.*, methyl lysine analogs (131) and enzymatical approach (132). The combination of MS with HDX has great potential to study organization and compaction of nucleosome and chromatin.

Dysregulation of histone PTMs leads to many human diseases such as cancer and neurological disorders. Exploiting epigenetic drugs is becoming increasingly attractive for therapeutic intervention. Beyond their potential as monotherapies, epigenetic drugs are now being tested with other therapies (*e.g.*, chemotherapy, radiation therapy, and immunotherapy) in clinical trials to minimize the risk of drug resistance and/or maximize the therapeutic efficacy (133). It has become evident that cancer cells are controlled by interconnected regulatory networks including signaling, metabolism, and epigenetics (12). MS-based multiomics approaches are useful tools to probe signaling pathways, chromatin status and metabolism, holding great promise to understand the complex regulatory mechanisms and promote future cotargeting strategies to block cancer development. To detangle these massive data sets from distinct layers, novel computational methods must be developed to integrate and analyze the multiomics data sets.

All in all, MS has proven to be an extremely useful tool that significantly contributes to the epigenetic field. Approaches from molecular biology, biochemistry, chemical biology, biophysics, and others coupled with MS have contributed enormously in recent years to comprehensive analysis of histone PTMs and their roles in regulation of chromatin functions. Despite the great advances, considerable work is needed to increase our understanding of the bigger picture of multiomics and identify epigenetic biomarker/drug associated with diseases.

## Conflict of interest

Authors declare no competing interests.
